# Complete Genome Sequence Analysis of *Bacillus subtilis* MC4-2 Strain That against Tobacco Black Shank Disease

**DOI:** 10.1155/2024/8846747

**Published:** 2024-03-25

**Authors:** Chunlan Shi, Shuquan Zeng, Xi Gao, Mehboob Hussain, Mingchuan He, Xurong Niu, Congcong Wei, Rui Yang, Mingxian Lan, Yonghui Xie, Zhijiang Wang, Guoxing Wu, Ping Tang

**Affiliations:** ^1^State Key Laboratory for Conservation and Utilization of Bio-Resources in Yunnan, Yunnan Agricultural University, Kunming 650201, China; ^2^Yunnan Tobacco Company Kunming Company, Kunming 650201, China

## Abstract

The MC4-2 bacterium strain was isolated and purified from the *Periplaneta americana* intestine as a biocontrol agent with good antagonistic effect against the pathogens of a soil-borne disease called tobacco black shank. The MC4-2 strain was found to have good broad-spectrum inhibition by plate stand-off test. Based on 16S rRNA and *gyrB* genes, ANI analysis, and other comparative genomics methods, it was determined that the MC4-2 strain was *Bacillus subtilis*. The complete genome sequence showed that the genome size was 4,076,630 bp, the average GC content was 43.78%, and the total number of CDSs was 4,207. Genomic prediction analysis revealed that a total of 145 genes were annotated by the CAZy, containing mainly GH and CE enzymes that break down carbohydrates such as glucose, chitin, starch, and alginate, and a large number of enzymes involved in glycosylation were present. A total of ten secondary metabolite clusters were predicted, six clusters of which were annotated as surfactin, bacillaene, fengycin, bacillibactin, subtilosin A, and bacilysin. The present investigation found the biological control mechanism of *B. subtilis* MC4-2, which provides a strong theoretical basis for the best use of this strain in biological control methods and provides a reference for the subsequent development of agents of this bacterium.

## 1. Introduction

Tobacco black shank is a soil-borne disease caused by *Phytophthora parasitica* var. *nicotianae* (*P. nicotianae*), which has the characteristics of rapid occurrence and great damage. It had severe impacts on the tobacco production in China and the rest of the world [1, 2]. At present, most of the scientific reports related to the chemical, agricultural, and biological control of tobacco black shank disease are reported. Among them, the use of chemical pesticides and the selection of disease-resistant varieties are the main control tactics [3–5]. With the strengthening of ecological protection, biological control methods that are safe, efficient, and environment friendly are gradually gaining the attention of the researchers.

*Bacillus* spp. are one of the most successful and widely studied biocontrol bacterial species in the world, and as a common dominant microbial population in soil, plants, and other environments, it had a strong resistance mechanism and potential for the disease suppression. As one of the model strains, *B. subtilis* has been widely studied for its biological control of plant diseases [6, 7]. The mechanisms of biocontrol of this bacterium are diverse and include competition, lysis, and induction of disease resistance in plants and promotion of plant growth. *B. subtilis* is capable of producing spores that are resistant to unfavorable environmental conditions such as heat, drought, organic solvents, and UV light, and it also produces bacteriocins, enzymes, lipopeptides, and other types of antimicrobial substances. In the course of searching for previous studies, the *B. subtilis* is able to colonize better in the interroot, surface, and interior of plants, the vast majority of available studies on the biocontrol effects of the bacterium have been related to aspects such as endophytes and soil interroot microorganisms, and there are fewer reports related to other sources. And even we found that biocontrol bacteria were mainly isolated from interroot and plant, and other sources of biocontrol bacteria are less frequently reported [8–10].

With the rapid development of modern molecular biology technology, especially sequencing technology, it is now easier and diversified to carry out the complete genome sequencing of bacteria [6, 11]. In a previous study, the insect-derived *B. subtilis* MC4-2 showed good plate and pot inhibition against the tobacco black shank pathogen *P. nicotianae*, but the mechanism of biological control is still unlearned [12]. It is also reported previously that Bacillus could produce a variety of secondary metabolites with antimicrobial effects, mainly including ribosomal and nonribosomal pathways [13, 14]. By analyzing the complete genome of bacteria, we can better understand the annotation of each such gene species, various carbohydrate enzymes, secondary metabolite species, and secreted proteins, which is beneficial for deeper investigation of the bacteriophage defense mechanism [8, 15, 16]. In this study, the MC4-2 strain differed from the other common source of biocontrol bacteria in that manner, because it is originated from the insect gut. The preliminary analysis of the complete genome of *B. subtilis* MC4-2 strain also shows its uniqueness in the research area of plant disease biocontrol bacteria of insect origin to a certain extent and provides more scientific basis for the subsequent research on the preparation of bacterial agents.

## 2. Materials and Methods

### 2.1. Biocontrol Strains and Culture

*B. subtilis* MC4-2strain was isolated from the intestinal tract of *P. americana* and stored in the laboratory of Pesticide Science, Plant Protection College of Yunnan Agricultural University, Kunming. Through preliminary experiments, we found that the MC4-2 strains have a good inhibitory effect on *P. nicotianae* which is effective against the soil-borne disease of tobacco black shank and conducted research on its fermentation conditions, etc. [12].

### 2.2. Bacteriostatic Test of This Biocontrol Agent

Using a plate confrontation test method, the sterilized hole punch (6 mm) was used to drill holes on the activated plate of plant disease pathogens, and then, sterile forceps were used to take a cake and inoculate it on the center of another new Potato Dextrose Agar medium (PDA). The single colony of MC4-2 strain isolated in advance was selected and inoculated at 2.5 cm around the PDA medium. After inoculation, the single colony was placed in a constant temperature incubator at 28°C for 3 d. Finally, the plate was taken out to calculate the bacteriostatic rate [12].

### 2.3. Experiment on Control of Indoor Pot Culture

After the soil was treated with antagonistic bacteria, tobacco seedlings were transplanted and inoculated with tobacco black shank pathogen. Pot tobacco seedlings without any soil treatment were used as blank control, and fungicide treatment was used as positive control. According to the classification standard of tobacco black shank disease, the number of disease strains and the incidence of disease were investigated, and the incidence rate, disease index, and control effect were calculated. There were three replicates for one treatment and nine pots of tobacco seedlings for one replicate [12].

### 2.4. Bacterial DNA Extraction

The MC4-2 strain was inoculated with liquid Luria Bertani medium (LB) and cultured for 24 h with the environment of 28°C, 180 r/min, using the Genomic DNA Purification Kit (Promega) and following the instructions [12].

### 2.5. Complete Genome Sequencing, Assembly, and Annotation

Bacterial DNA was extracted and sent to Shanghai Meiji Biomedical Technology Co., Ltd. for complete genome sequencing and assembly using second-generation and third-generation, i.e., Illumina Hiseq and PacBio sequencing [17, 18]. The data of ANI, covariance, and pangenomic analysis was analyzed on the online tool of Majorbio Cloud Platform [19].

Prediction of rRNA, tRNA, and CDS was performed by using Barrnap software, tRNA scan-SE v2.0 software, Glimmer, Gene MarkS, and Prodigal [20, 21]. Circle mapping was performed using CGView v2.0 software [22]. COG, GO, and KEGG annotation was performed using Blast2go v2.5 (http://www.blast2go.com), EggNOG Database (http://eggnog.embl.de), KEGG Database (http://www.genome.jp/kegg), etc. [23, 24]. CAZy annotations were compared using the Carbohydrate-Active Enzyme Database (http://www.cazy.org), and the antiSMASH v6.0 (http://antismash.secondarymetabolites.org) tool was used for the secondary metabolite gene decision cluster prediction [25]. The above bioinformatics work about whole genome annotation, predictive analysis, and mapping was assisted by Shanghai Meiji Biomedical Technology Co., Ltd.

### 2.6. Formulation of the Medium Used

Potato Dextrose Agar medium (PDA): potatoes 200 g, glucose 20 g, agar 15~20 g, distilled water 1,000 mL. Luria Bertani medium (LB): tryptone 10 g, yeast extract 5 g, NaCl 10 g, agar 15~20 g, distilled water 1,000 mL.

## 3. Result

### 3.1. The Bacteriostatic Effect of MC4-2 Strain

**Figure 1:**
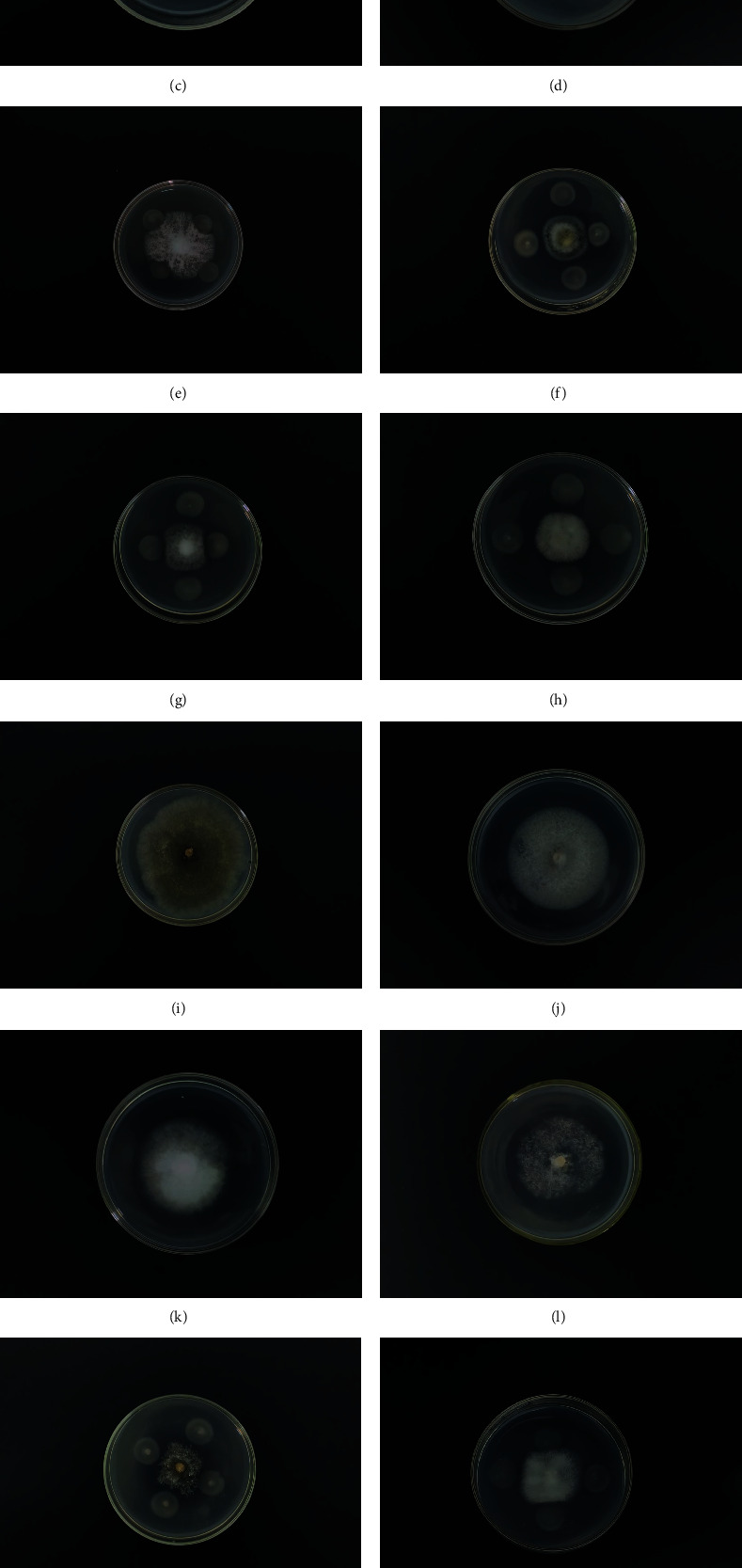
Inhibition of different pathogens by MC4-2 strain. (a) Tobacco root rot disease (*F. oxysporum*). (b) Potato early blight disease (*A. solani*). (c) Round spot disease of *Panax notoginseng* (*M. acerina*). (d) Cucumber target spot disease (*C. cassiicola*). (i) Tobacco target spot (*R. solani*). (j) Coffee anthracnose (*C. gloeosporioides*). (k) Citrus brown spot disease (*A. alternata*). (l) Tobacco black shank disease (*P. parasitica*). (e–h, m–p) Results of flat plate stand-off corresponding to (a–d, i–l).

By means of a plate stand-off test, we found that the MC4-2 strain had broad-spectrum antibacterial effect against plant pathogens that cause diseases (Figure 1), such as *Rhizoctonia solani*, *Alternaria alternata*, *Corynespora cassiicola*, *Pestalotiopsis eriobortfolia*, *P. nicotianae*, *Alternaria solani*, *Mycocentrospora acerina*, *Fusariumoxysporum*, and *Colletotrichum gloeosporioides*.

### 3.2. Indoor Control Efficacy of Strain MC4-2 against Tobacco Black Shank Disease

The results of the indoor pot experiment are shown in Table 1. The control effect of strain MC4-2 on tobacco black shank disease can reach 51.20%, and the control effect of 64% oxadixyl mancozeb on tobacco black shank disease can reach 70.56%.

### 3.3. Determination of the Taxonomic Status of Strain MC4-2

#### 3.3.1. Evolutionary Tree of MC4-2 Strain

**Figure 2:**
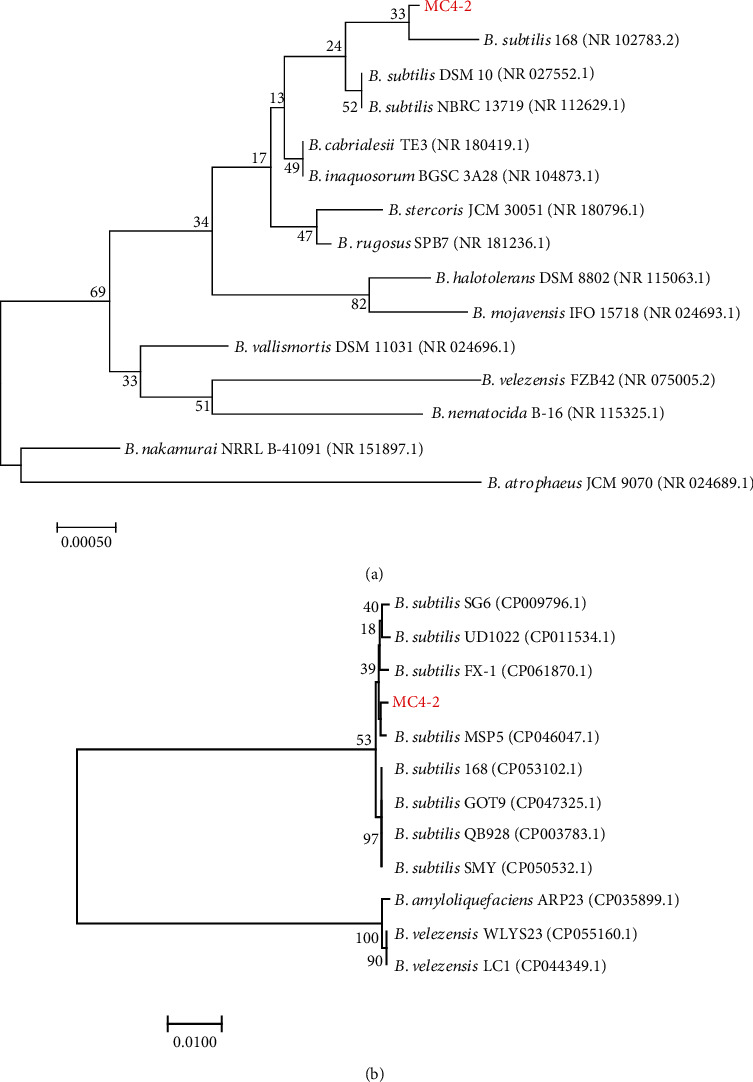
Evolutionary tree of strains constructed based on (a) 16S rRNA gene and (b) *gyrB* gene.

The 16S rRNA gene sequences were intercepted in the complete genome of MC4-2 strain and then BLAST compared in GeneBank. The MC4-2 strain was similar to sequence of *B. subtilis* 168 and was in the same branch (Figure 2(a)). Nevertheless, the 16S rRNA gene was used for identification at the genus level commonly. Therefore, we could identify the MC4-2 strain as *Bacillus* spp., by the 16S rRNA gene.

The *gyrB* gene for MC4-2 strain has high homology with *B. subtilis* (Figure 2(b)). Since the *gyrB* gene is a more accurate method compared to the 16S rRNA gene, by correlating this relationship with the evolutionary relationship of Figure 2, we can infer that MC4-2 strain was *B. subtilis*.

#### 3.3.2. ANI Analysis of Different *Bacillus* spp. Strains

**Figure 3:**
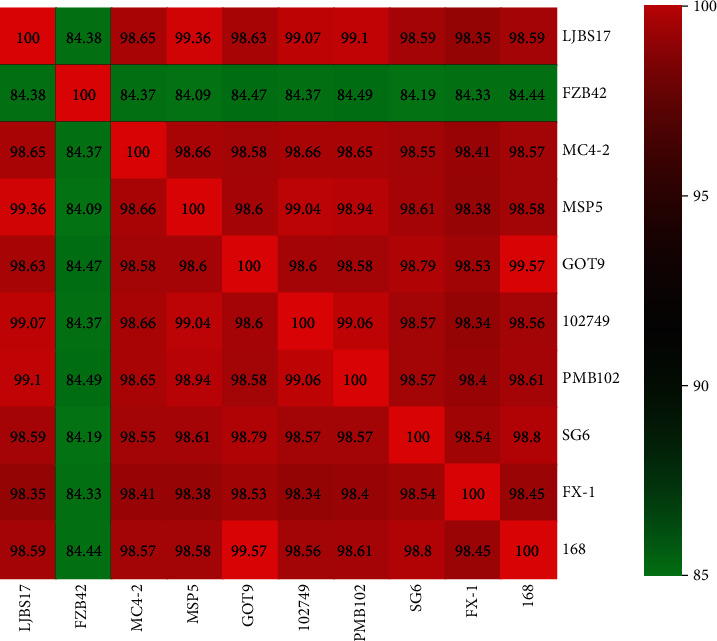
Heat map constructed based on the average nucleotide identity of 10 strains.

We analyzed the complete genomes of several strains with high similarity in GenBank after BLAST against strain MC4-2 and found that all strains showed high similarity (ANI values > 95%) to each other (*B. subtilis*), except for *B. velezensis* FZB42 (Figure 3).

#### 3.3.3. Analysis of Covariance between MC4-2 Strain and Reference Strain

**Figure 4:**
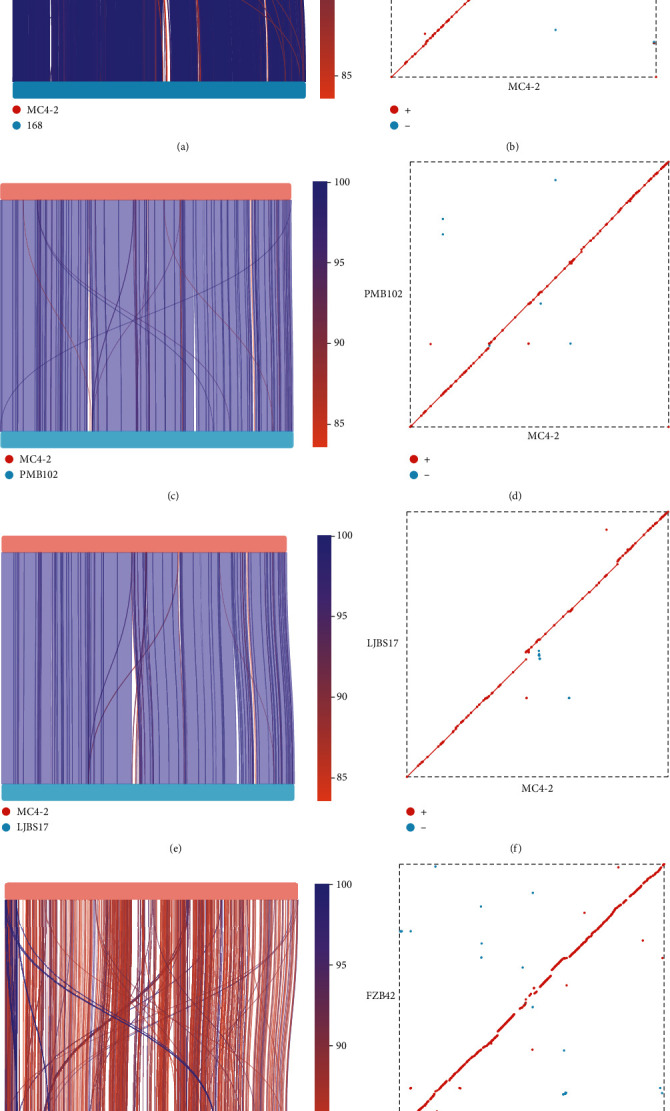
Results of interstrain covariance analysis. (a) Analysis of covariance with MC4-2 and 168 strains. (b) Linear fitting of MC4-2 and 168 strains. (c) Analysis of covariance with MC4-2 and PMB102 strains. (d) Linear fitting of MC4-2 and PMB102 strains. (e) Analysis of covariance with MC4-2 and LJBS17 strains. (f) Linear fitting of MC4-2 and LJBS17 strains. (g) Analysis of covariance with MC4-2 and FZB42 strains. (h) Linear fitting of MC4-2 and FZB42 strains.

From the above results, we performed a covariance analysis to MC4-2 strain with the different sources of *B. subtilis* 168 (model strains), *B. subtilis* PMB102 (tomato leaves), and *B. subtilis* LJBS17 (grape rhizosphere soil) and found that the nucleotide sequences were highly similar which is a good linear relationship between them, but there were also some genomic rearrangements such as flips and translocations (Figure 4). As a reference strain, 168 PMB102 and LJBS17 strains are both *B. subtilis*, so MC4-2 strain has a good degree of covariance with both of them (Figures 4(a), 4(c), and 4(e)). However, we have taken MC4-2 strain as a comparison with the FZB42 strain; the result showed that collinearity exists, but the degree of collinearity was lower than other comparisons (Figure 4(e)). This suggests that complete genome sequence between different species of *Bacillus* spp. was greater than those which were between the same species.

### 3.4. Overview of the Complete Genome of MC4-2 Strain

**Figure 5:**
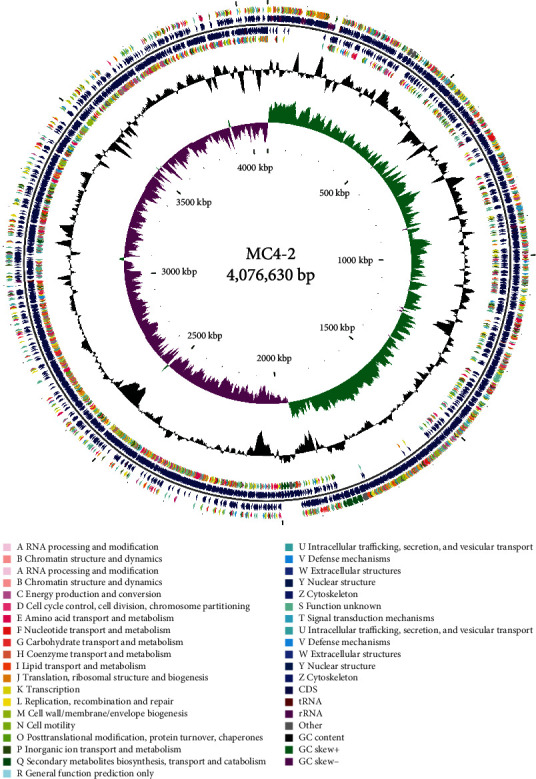
GCView map of MC4-2 strain. Each circle of the GCview circle from outside to inside represents the following meanings. (A) The first and fourth circles are the protein-coding sequence (CDS) on the positive and negative chains, respectively, and different colors correspond to different COG annotation results. (B) The second and third circles represent the protein-coding sequence (CDS), tRNA gene, and rRNA gene on the positive and negative chains, respectively. (C) The fifth circle represents the GC content of the genome sequence, and the outward and inward parts represent the GC content of the region higher or lower than the average GC content of the complete genome sequence, respectively. (D) The sixth circle represents the GC skew value, and the specific algorithm is G − C/G + C. Generally, the lead chain (GC skew and GT; 0) and trailing lag chain (GC skew and lt; 0) can also assist in judging the replication starting point (minimum cumulative offset) and end point (maximum cumulative offset). (E) The innermost circle indicates the size of the entire genome.

The complete genome size of MC4-2 strain was 4,076,630 bp, the average GC content was 43.78%, and the total number of protein-coding genes was 4,207 (Table 2). The total length of all coding genes was 3,584,916 bp, and the average length of coding genes was 852 bp. It contains 34 tandem repeats with a total length of 24,966 bp, accounting for 70% of the genome, and the size was 18-282 bp. It contained 85 tRNA genes, 20 tRNA types, and 30 rRNA genes, including 10 16S rRNA, 10 23S rRNA, and 10 5S rRNA (Figure 5).

### 3.5. Pan and Core Genomic Analysis of Different *Bacillus* spp. Strains

**Figure 6:**
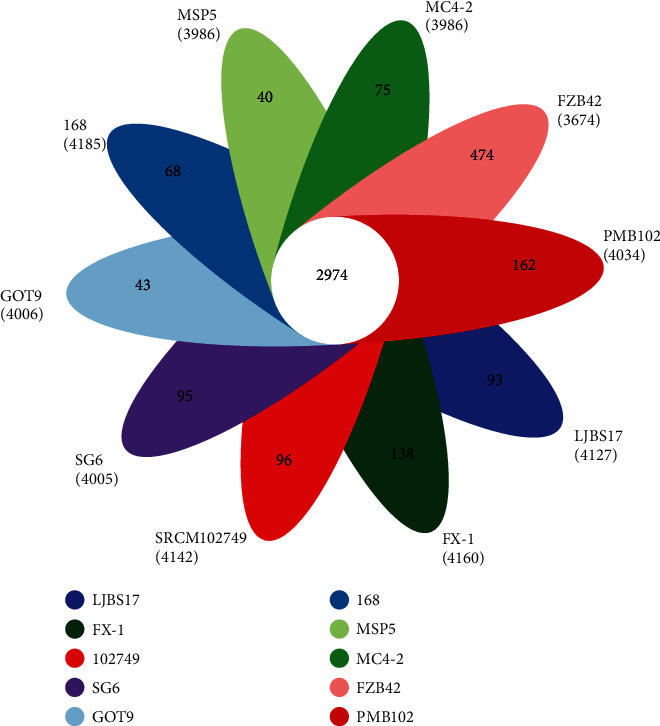
A Venn diagram of homologous genes.

In order to clarify the relationship of homologous genes between the MC4-2 strain and other similar strains, the pan genome and core genome of 10 *Bacillus* spp. strains were performed, and a Venn diagram was designed. There were 5,786 pan genomes, 2,974 core genomes, and 1,284 unique genes (Figure 6). The *B*. *subtilis* MC4-2 had 3,986 homologous coding genes, and it had 75 unique CDSs (core genes) compared to other strains, and the number of CDSs shared by the 10 strains was 2,974 which were core genes. The unique genes of *B. velezensis* FZB42 were significantly higher than other *B. subtilis*, because it is more distantly related to other strains.

### 3.6. Genome Function Annotation

The NR, Swiss-Prot, Pfam, COG, GO, and KEGG databases were used to compare the protein sequence of the predicted gene with each functional database to obtain the functional annotation information in the database (Table 3). According to the results of gene annotation and prediction, we summarized apart of genes related to inducing plant disease resistance, growth promotion, colonization, and signal transduction in strain MC4-2 (Table 4).

#### 3.6.1. COG Notes

**Figure 7:**
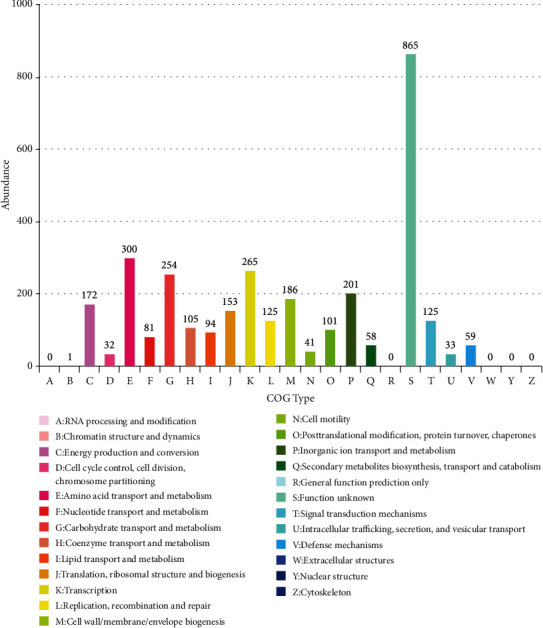
COG annotation results of MC4-2 strain.

COG annotation was performed on the protein-coding genes with biological functions in the genome of MC4-2 strain, and it was found that a total of 3,202 protein-coding genes were annotated, and they were divided into 26 types from A-Z (Figure 7). Among them, there were 865 functions unknown, with the largest number of annotated genes, followed by 300 amino acid transport and metabolism (E), 265 transcription (K), and 254 carbohydrate transport and metabolism (G). There were 201 for inorganic ion transport and metabolism (P), 186 for cell wall/membrane/envelope biogenesis (M), 172 for energy production and conversion (B), and other COG types. The above six categories accounted for 9.37%, 8.28%,7.93%, 6.28%, 5.81%, and 5.37% of the total, respectively.

#### 3.6.2. GO Annotation

**Figure 8:**
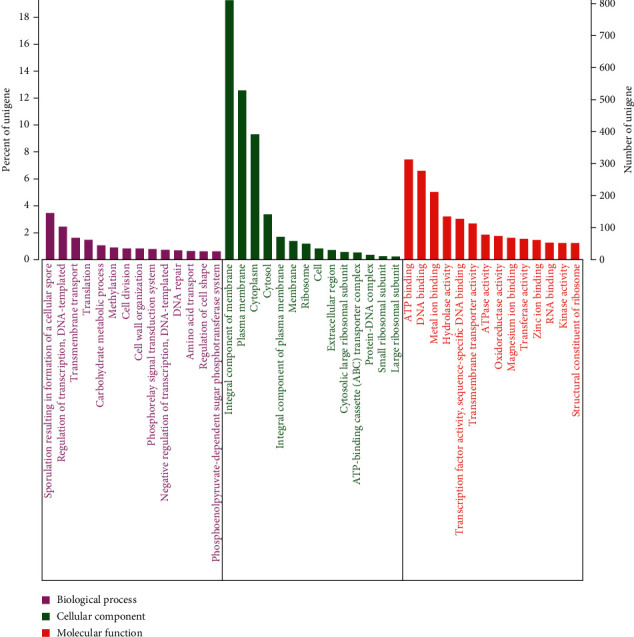
GO annotation results of MC4-2 strain.

There are 2721 genes annotated, through the GO annotation, and it divided the functionality into three parts of biological process, cellular component, and molecular function, which the second part accounts for the largest proportion (Figure 8). In the part of cellular component, the genes of integral component of membrane have the largest proportion (811), followed by plasma membrane (529) and cytoplasm (392). In the portion of biological process, the genes (CDS) of sporulation resulting in the formation of a cellular spore (146) accounted for the largest. It could reflect that the MC4-2 strain acts as a bacteriostatic agent by producing spores and forming biofilms principally. Meanwhile, in the part of molecular function, binding of DNA (313), ATP (278), and metal ion (211) was the main content of genes. This shows that strain genes were expressed more in proliferation, generating energy and metabolism.

#### 3.6.3. KEGG Notes

**Figure 9:**
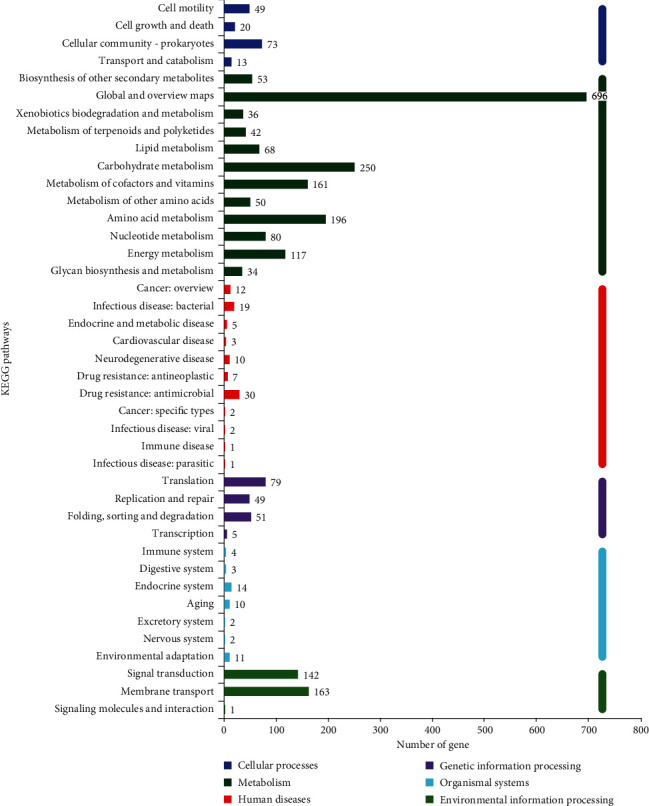
Annotated graph of MC4-2 strain KEGG.

The whole genome sequence of MC4-2 strain was compared with the KEGG database, and a total of 2330 genes were annotated (Figure 9). The KEGG annotation classifies functional genes into six systems: cellular processes, metabolism, human diseases, genetic information processing, organismal systems, and environmental information processing. A total of 1783 genes were expressed in the metabolic system, with the most annotated results for global and overview maps (696), followed by 250, 196, and 161 genes for carbohydrate, amino acid and coenzyme factors, and vitamin metabolism, respectively. This indicates that MC4-2 strain genes are more expressed in such metabolic activities, which is also related to the ability of the strain to produce a large number of active substances such as proteins and enzymes. In addition, 306 genes were annotated in the environmental information processing system, with 46.41% and 53.27% of signal transduction and membrane transport, respectively. The rest of the systems were annotated with a smaller number of genes.

### 3.7. Metabolic System Analysis

#### 3.7.1. CAZy Carbohydrate-Active Enzyme Analysis

The number of CAZy-annotated genes was obtained by comparing the genes related to MC4-2 strain with the Carbohydrate-Active Enzyme Database (Table 5). Based on the similarity of amino acid sequences in the protein structural domains, the carbohydrate-active enzymes of different species origin could be classified into glycoside hydrolases (GH), glycosyl transferases (GT), polysaccharide lyases (PL), carbohydrate esterases (CE), carbohydrate-binding modules (CBM), auxiliary activities (AA), and other six protein families.

From the carbohydrate-active enzyme species annotated by MC4-2 strain, all the above six protein types were included, among which glycoside hydrolase (GH) had the largest number of genes (55) and contained hydrolases such as *β*-glucosidase, *α*-amylase, *α*-glucanase, gibberellins, glucanase, and lysozyme. Glycosyl transferase (GT) had 46 genes, mainly containing UDP-glucuronosyltransferase, UDP-N-acetylglucosamine-like *β*-N-acetylglucosaminyltransferase, and other enzymes. Carbohydrate esterase (CE) with 30 genes mainly contains acetyl xylan esterase, pectin acetyl esterase, aromatic esterase, rhamnogalacturonan acetyl esterase, and other enzymes. The polysaccharide lyase (PL) and auxiliary oxidoreductase (AA) contained 9 and 7 genes, respectively, and mainly contained rhamnogalacturonan endonuclease, 1,4-benzoquinone reductase, and cellobiose dehydrogenase, respectively. Secondly, the carbohydrate-binding module (CBM) had three genes annotated and was mainly associated with enzymes that cleave gibberellins or peptidoglycans. The above annotation of MC4-2 strain CAZy showed the presence of enzymes capable of encoding the production of hydrolases and esterases that break down carbohydrates, such as glucose, gibberellins, starch, and alginate, and the presence of a large number of enzymes involved in glycosylation, an important modifying effect of the enzymes on the protein.

#### 3.7.2. Analysis of Secondary Metabolite Synthesis Gene Clusters

Microbial secondary metabolism is when the growth of microorganisms reaches a certain growth period (usually a stable period), it will use primary metabolites as precursors and undergo a series of metabolic processes such as polymerization and assembly to synthesize some that have no clear function for their own life activities. Substances are, namely, secondary metabolites. Secondary metabolites are generally controlled by multiple genes, and their coding genes usually exist in clusters in the genome, encoding complex enzymes with multiple functions. This gene cluster is the secondary metabolite synthesis gene cluster.

The antiSMASH software was used to predict the secondary metabolite synthesis gene cluster of the sample. The complete genome of strain MC4-2 was imported into the antiSMASH online tool (Table 6). Ten gene decision clusters were successfully compared, and six clusters were able to find similar clusters in NCBI. There are around 82% similarity between Cluster 1 and surfactin of MIBiG registration number BGC0000433, 100% similarity between Cluster 3 and bacillaene of BGC0001089, 100% similarity between Cluster 4 and fengycin of BGC0001095, and 100% similarity between Cluster 4 and fengycin of BGC0001095. Cluster 4 was 100% similar to fengycin from BGC0001095, Cluster 7 was 100% similar to bacillibactin from BGC0000309, and Cluster 9 was 100% similar to bacillibactin A from BGC0000602. Subtilosin A of BGC0000602 was 100% similar. In addition, Cluster 5 and Cluster 6 were terpene and curcumin (T3PKS), respectively, which also have antibacterial effects, but the specific comparison results were not yet cleared, and further studies such as simple compound isolation may be needed. Comparative NCBI searches revealed that the secondary metabolite gene determinant clusters of *B. subtilis* strain MC4-2 were similar to those of *B. velezensis* FZB42 (BGC0000433, BGC0001089, BGC0001095, and BGC0001184), *B. subtilis* 168 (BGC0000309), and *B. subtilis* ATCC 6633 (BGC0000602) gene clusters.

## 4. Discussion

With the development of research, *B. subtilis* has gradually fully fledged and has shown a good antibacterial effect in the biocontrol of crop diseases. With the whole genome sequencing of the first strain of *B. subtilis* 168 [6], a large number of biological technology studies have been carried out on this bacterium, because this bacterium plays an important role not only in biocontrol but also in industry and food, etc. [40, 41].

The inhibition rate of MC4-2 strain against *P. nicotianae* that is the pathogen of tobacco black shank disease was more than 60% [12]. In addition, strain MC4-2 showed 51.20% effectiveness against tobacco black shank disease in laboratory experiments. According to the antibacterial test, the MC4-2 strain has a wide antibacterial spectrum, which has a good antibacterial effect on the fungal pathogens of some genera of the Deuteromycota and also has a good antibacterial effect on some pathogens of the Oomycota and Ascomycota. In this study, the MC4-2 strain was isolated from the intestinal tract of *P. americana*, which is different from the common source. Due to the particularity of its living environment, in order to adapt to the environment and coevolution, there must be a large number of symbiotic bacteria in its body to resist the pathogens in the environment. Some studies have isolated many bacteria that inhibit pathogens from the intestinal tract of *P. americana* [42]. Moreover, there is rich microbial diversity in the intestinal tract of this specie [43].

On account of the previous studies, it was shown that the 16S rRNA gene alone could not achieve the best results for the strain identification [44]. Therefore, in this study, in order to clarify the taxonomic status of strain MC4-2, we constructed the evolutionary tree of its 16S rRNA and *gyrB* gene and found that the strain was *B. subtilis*. However, the evolutionary tree construction alone may not be enough to completely prove the taxonomic status of this bacterium. To test whether this result was accurate enough, ANI and collinearity analyses were performed on the whole genome of the bacterium, which the results also showed that the MC4-2 strain was very similar to *B. subtilis*. It is worth mentioning that the collinearity analysis between strain MC4-2 and biocontrol *B. subtilis* isolated from tomato leaves and grape rhizosphere soil showed a high degree of fit, as did the similarity between the engineered 168 strains. This indicates that even *B. subtilis* of different origin has great similarities in evolutionary relationships and genetic composition.

Carbohydrates play an important role in many biological functions, and a lot of meaningful biological information can be obtained by studying carbohydrate-related enzymes. There were 145 coding genes annotated by CAZy in strain MC4-2, including *β*-glucosidase, *α*-amylase, *α*-trehalase, chitinase, and other hydrolases (*bglA*, *amyA*, *mapA*, and *yaaH* gene). In the original tests on the physiological and biochemical characteristics of MC4-2, it also showed positive reactions to starch hydrolysis and glucose hydrolysis [12]. In addition to hydrolases, 46 genes encoding glycosyl transferases have been annotated in this strain, and studies have shown that these enzymes are closely related to microbial antibiotics and other production [45, 46].

Among the annotated genes, many genes involved in promoting plant growth and disease prevention were also predicted. We found that the MC4-2 strain contained *trpA*, *trpB*, *trpC*, and *trpS* genes, which were involved in the regulation of the biosynthesis of IAA, a plant growth hormone, and had a direct relationship with the promotion of plant growth, and some genes involved in the synthesis of important amino acids in plants, such as *ilvB*, *ilvH*, and *alsD*. Meanwhile, in addition to the direct promotion of plants, the decomposition of trace elements in soil is also a way to promote plant growth [47, 48]. For example, *phoD*, *phoA*, *phoE*, and *ktrA* genes related to phosphorus and potassium decomposing protein were found in MC4-2 strain. These results indicated that *B. subtilis* MC4-2 isolated from the intestinal tract of *P. americana* promotes plant growth as well as the plant growth-promoting rhizobacteria (PGPR) [16, 27, 49].

Moreover, several studies have shown that bacteria usually need to adsorb to the plant roots or colonize the plant, and normally, we consider these bacteria to be beneficial. The ability of microorganisms to successfully colonize the surface or interior of plants is critical to the ability to promote plant growth and health [50]. These bacteria can swim through flagella to reach the plant surface or enter the plant and dominate and then produce some enzymes, proteins, and other active substances that interact with the plant [50–52]. Also, the formation and function of biofilms are highly related to bacterial colonization. Not only that, the secretion of various proteins is likewise associated with biofilms, such as *tasA*, *slrR*, *sinR*, and other genes.

The antiSMASH online tool was used to predict the secondary metabolite gene clusters of strain MC4-2, and ten clusters were annotated, six of which were known gene clusters and four were unknown gene clusters. The six gene clusters encode surfactin, bacillaene, fengycin, bacillibactin, subtilosin A, bacilysin, and other antimicrobial substances. Surfactant plays a great role in the formation and movement of cell membranes and also has antibacterial, antimycoplasma, and antiviral activities, which are the most important antibacterial active substances [53–55]. Because of its unique amphiphilic structure, it is an excellent biosurfactant. Through experiments on *Aedes aegypti*, *Anopheles aegypti*, and *Culex aegypti*, Geetha and Manonmani [56] found that surfactant also had the potential to eliminate the activity of mosquitoes. Bacillaene is a polyene antibiotic that plays a role in bacteria and fungi by suppressing protein synthesis. Fungicin has a good inhibitory effect on fungi, especially filamentous fungi [57]. Most of the biocontrol Bacillus can produce ferricarriers, which have a high affinity for Fe^3+^ and further chelate ferrivalent ions to reduce the ferrivalent ions in the soil, thus inhibiting the growth of pathogenic bacteria [58]. Subtilosin A can inhibit the growth of pathogenic bacteria mainly by changing the permeability of cell membrane, which has an inhibitory effect on many Gram-negative and Gram-positive bacteria [59, 60]. Among the secondary metabolites, bacillomycin is the only compound that can inhibit aflatoxin [61]. Gao et al. [62] isolated *B. subtilis* VD18R19 from the root system of vanilla, conducted whole genome sequencing, and found that the strain contained six gene clusters of antimicrobial secondary metabolites synthesis, coding surfactin, plipastatin, bacillibactin, bacilysin, bacillaene, subtilosin A, and other antibacterial substances; Wang et al. [63] sequenced the whole genome of *B. subtilis* Bs-916. It was found that eight NRPS/PKS gene clusters encoding surfactin and bacillomycin detected L, fengycin, bacillibactin, bacilysin/anticapsin, macrolactin, bacillaene, difficidin, and other substances with the ability to secrete a variety of antibacterial substances. Qi et al. [64] found seven gene clusters in *B. subtilis* BS-6 through whole genome sequencing. These gene clusters encode substances associated with bioantibiotic synthesis such as subtilin, subtilosin A, surfactin, bacillibactin, bacillaene, mycosubtilin, and rhizocticin. Although they are all *B. subtilis*, genome-wide analysis showed that the gene clusters encode different substances, which is most likely due to the different origins of several strains of *B. subtilis*.

## 5. Conclusion

The MC4-2 strain was isolated from the intestinal tract of *P. americana* and showed good inhibition against *P.nicotianae* in plates and potted plants, with a broad antibacterial spectrum. And 53.78% of the control effect of tobacco black shank disease is in an indoor control experiment. The 16S rRNA and *gyrB* gene evolutionary trees were constructed, and the whole genome of MC4-2 was analyzed by ANI and collinearity, which proved that the strain was *B. subtilis* to a large extent. By analyzing the whole genome and mining hidden information, further understanding of each gene species, various carbohydrate enzymes, secondary metabolite species, and secreted proteins will be beneficial to further study the biological control mechanism of bacteria. The whole genome sequence showed that the genome size was 4,076,630 bp, the average GC content was 43.78%, and the total number of CDSs was 4,207. Genomic prediction analysis showed that CAZy annotated a total of 145 genes, mainly containing GH and CE enzymes that decompose carbohydrates such as glucose, chitin, starch, and alginate, as well as a large number of enzymes involved in glycosylation. *trpA*, *trpB*, *trpC*, and *trpS* genes are involved in the regulation of IAA biosynthesis. *phoD*, *phoA*, *phoE*, and *ktrA* genes were associated with phospho-potassium decomposition proteins. A total of ten secondary metabolite clusters were predicted. antiSMASH online tool was used to predict the secondary metabolite gene clusters of MC4-2 strain, and 10 clusters were annotated. Four clusters were not found, and five clusters had highly similar gene determinants. Six clusters were annotated as surfactin, bacillaene, fengycin, bacillibactin, subtilosin A, and bacilysin. This study revealed the biological control mechanism of *B. subtilis* MC4-2 and provided a strong theoretical basis for the subsequent research and the development of biocontrol agents.

## Figures and Tables

**Table 1:** Results of indoor control effect of strain MC4-2 on tobacco black shank.

Treatment	Disease index	Average control effect (%)
MC4-2 fermentation broth	37.07 ± 0.52a	51.20 ± 0.65b
64% oxadixyl mancozeb	22.36 ± 0.49b	70.56 ± 0.46a
CK	75.97 ± 0.81c	—

Note: Different letters after the data in the same column indicate significant differences.

**Table 2:** The complete genome overview of MC4-2 strain and others.

Items	*B. subtilis* MC4-2	*B. subtilis* 168 [6]	*B. subtilis* ATCC 13952 [26]	*B. subtilis* XF-1 [27]	*B. amyloliquefaciens* BS-3 [28]
Genome size (bp)	4,076,630	4,215,606	3,876,276	4,061,186	3,870,130
G + C content (%)	43.8	43.5	45.8	43.8	46.9
Protein-coding sequences	4,207	4,255	3,852	3,853	4,161
Average CDS size (bp)	852	872	877	–	832
Number of tRNAs	85	86	72	77	92

–: no data found.

**Table 3:** The annotation information of MC4-2 strain.

Type	Gene no.	Annotation rate (%)
NR	4207	100
Swiss-Prot	3851	91.5
Pfam	3535	84.0
COG	3202	76.1
GO	2721	64.7
KEGG	2330	55.4

**Table 4:** Prediction of genes related to induction of plant disease resistance, growth promotion, colonization, and signal transduction in strain MC4-2.

Gene name	Protein description	Reference
*trpA*	Tryptophan synthase subunit alpha	[16, 29]
*trpB*	Tryptophan synthase subunit beta	[16, 29]
*trpC*	Indole-3-glycerol phosphate synthase	[16, 30]
*trpS*	Tryptophanyl-tRNA synthetase	[27]
*ilvB*	Acetolactate synthase large subunit	[27]
*ilvH*	Acetolactate synthase small subunit	[27]
*alsD*	Acetolactate decarboxylase	[16, 27]
*phoD*	Alkaline phosphatase	[31]
*phoA*	Alkaline phosphatase	[32]
*phoE*	Phosphatase	[33]
*ktrA*	Potassium uptake protein	[27]
*tasA*	Biofilm matrix protein	[34]
*mstX*	Biofilm formation protein	[35]
*epsG*	Biofilm exopolysaccharide biosynthesis protein	[36]
*slrR*	Biofilm formation regulator	[37]
*sinR*	Master regulator for biofilm formation	[37]
*flgB*	Flagellar basal body rod protein	[38]
*flgC*		
*flgE*		
*flgD*	Flagellar hook assembly protein	[38]
*flgK*	Flagellar hook-associated protein	[38]
*flgK*		
*flgM*	Flagellar biosynthesis antisigma factor	[38]
*motA*	Flagellar motor stator protein	[39]
*motB*	Flagellar motor protein	[39]
*luxS*	S-ribosyl homocysteine lyase	[16]

**Table 5:** Number of annotated genes of MC4-2 strain CAZy.

CAZy type	Partial gene name	Gene no.
Glycoside hydrolases	*bglA amyA ntdC yteR xynAC nagZ gmuG lacZ lplD yaaH abfA mapA*	55
Glycosyl transferases	*rapA ugtP bshA*	46
Carbohydrate esterases	*menH pnbA bshB pdaA nagA glgA*	30
Polysaccharide lyases	*yesW yesX*	9
Auxiliary activities	*cotA wrbA glcD thiO*	7
Carbohydrate-binding modules	*exlX*	3

**Table 6:** Secondary metabolite gene prediction.

Cluster ID	Type	Partial gene name	MIBiG accession	Most similar cluster	Similarity (%)	Gene no.	Nucleotide length (bp)
Cluster 1	Assimilatory nitrate reductase electron transfer subunit NasB	*srfAA* *srfAB* *srfAC*	BGC0000433	Surfactin	82	48	62963

Cluster 2	Multispecies: germination protein GerPC	−	−	−	−	23	20396

Cluster 3	transAT-PKS	*pksC* *pksD* *pksE* *pksJ* *pksL* *pksM* *pksN* *pksR* *pksS*	BGC0001089	Bacillaene	100	58	114777

Cluster 4	DNA topoisomerase IV subunit A	*ppsA* *ppsB* *ppsC* *ppsD* *ppsE*	BGC0001095	Fengycin	100	43	82279

Cluster 5	Terpene	−	−	−	−	23	21899

Cluster 6	T3PKS	−	−	−	−	47	41098

Cluster 7	NRPS	*entB* *entE* *dhbF*	BGC0000309	Bacillibactin	100	47	49742

Cluster 8	CDPS	−	−	−	−	19	20747

Cluster 9	Head to tail	*narG* *narH* *narK* *narS* *rapF*	BGC0000602	Subtilosin A	100	21	21612

Cluster 10	Other	*bacD*	BGC0001184	Bacilysin	100	44	41419

–: no data found.

## Data Availability

The authors will supply the relevant data in response to reasonable requests.
